# Prevalence of *Toxoplasma gondii* ‐like oocyst shedding in feral and owned cats in Damascus, Syria

**DOI:** 10.1111/jvim.16683

**Published:** 2023-04-18

**Authors:** Mohammad Taher Ismail, Abeer Al‐Kafri

**Affiliations:** ^1^ Department of Microbiology and Biochemistry, Faculty of Pharmacy Arab International University (AIU) Ghabagheb Syria

**Keywords:** cats, oocysts, Syria, *Toxoplasma gondii*‐like

## Abstract

**Background:**

The incidence of toxoplasmosis in humans in Syria indicates an increase in the number of infections with this disease. Cats are the only definitive host of *Toxoplasma gondii* and excrete environmentally resistant oocysts in their feces.

**Objectives:**

Estimate the prevalence of *T. gondii*‐like oocyst shedding in the cat population in Damascus, Syria.

**Animals:**

One‐hundred domestic cats.

**Methods:**

One‐hundred fecal samples from cats (68 feral cats and 32 owned cats) were collected in Damascus between October and December 2017 and examined for *T. gondii*‐like oocysts by direct microscopic examination using Sheather's sugar flotation procedure.

**Results:**

Examination of the samples showed that 36% (36/100) of the cats were shedding *T. gondii*‐like oocysts. Sporulated or unsporulated oocysts morphologically consistent with *T. gondii* were detected in 38.2% (26/68) of the samples collected from feral cats and in 31.3% (10/32) of the samples collected from client‐owned cats.

**Conclusion:**

The clinical importance of Toxoplasmosis in humans lies in the transmission of Toxoplasma to the fetus especially in the first trimester, resulting in severe clinical symptoms in the infant and leading to spontaneous abortion, stillbirth or other serious health problems and severe sequelae (e.g., mental retardation, blindness, hearing, and neurological disorders). Our results showed higher prevalence in Syria than in Lebanon. High amounts of *T. gondii*‐like oocyst shedding were detected in both feral and client‐owned cats in Damascus, emphasizing the importance of further research to understand *T. gondii* infection in people and animals in this region.

## 1 INTRODUCTION

Toxoplasmosis is a zoonotic disease caused by *Toxoplasma gondii*.^1^ *Toxoplasma gondii* is an obligate intracellular parasitic protozoan with a worldwide distribution,^2^ and is an apicomplexan protozoan parasite that infects birds and mammals, including humans. Although infections often are asymptomatic, *T. gondii* can cause serious disease and death in humans and animals.^3^, ^4^

Toxoplasmosis is widespread, and present in every country. Latent infection with *T. gondii* is among the most prevalent of human infections. It is estimated that one‐third of the world's human population is infected with this parasite. Seropositivity rates range from <10% to >90% in human populations in different areas of the world, which varies with cultural and eating habits.^5^ Studies of toxoplasmosis in humans in Damascus, Syria indicate that there has been an increase in the incidence of this disease, with incidence ranging between 30% and 89.7%.^6^, ^7^, ^8^

Transmission of toxoplasmosis to humans occurs by eating raw meat containing cysts of Toxoplasma, through food or water contaminated with oocysts excreted by cats,^9^ or congenitally via transplacental transmission (congenital toxoplasmosis). If transmission of the parasites to the fetus occurs in the first trimester, it results in the most severe clinical symptoms in the infant leading to spontaneous abortion, stillbirth or other serious health problems and severe sequelae (eg, mental retardation, blindness, hearing, and neurological disorders).^10^ In addition, infection with *T. gondii* is increasingly being recognized as a problem in non‐pregnant, immunocompetent adults, where acute infection may lead to impaired eyesight.^11^

Cats are reservoirs for several parasites, some of which are responsible for serious zoonotic diseases. They are the only definitive host of *T. gondii* and excrete environmentally‐resistant oocysts in their feces.^12^ Therefore, a high risk of Toxoplasma infection exists in humans living in cities with high densities of feral and owned cats.^13^

Cats begin to shed oocysts as early as 3 to 8 days after being fed tissue containing cysts. However, oocysts are less infectious and pathogenic to cats than to intermediate hosts.^14^ Ingesting oocysts in water, soil or feed is probably the most common route for *T. gondii* infection in non‐carnivorous mammals and birds. Seroprevalence of *T. gondii* can be high in meat‐producing animals around the world (up to 100%), but infection is more common in certain livestock species including sheep and goats.^15^

Interest in detecting *T. gondii* oocysts in the environment is emerging because of recent outbreaks of waterborne toxoplasmosis in humans. Oocysts can survive various inactivation procedures especially those using chemical reagents,^16^ and remain viable in water even after exposure to aqueous 2% sulfuric acid for at least 18 months at 4°C. They also resist detergents or disinfectant solutions such as sodium hypochlorite. Drinking water treatment plants using chlorination as the sole method of disinfection could therefore supply water containing infective oocysts.^17^

In humans, the percentage of infections caused by oocysts remains undetermined.^18^ *Toxoplasma gondii* infection seems strongly associated with soil contact,^19^ contributing up to 17% of infections among pregnant women in Europe,^18^ which emphasizes the key role of the environment as a source of infection. Oocysts also can be transmitted to humans by eating contaminated vegetables. A study in Italy showed that in ready‐to‐eat packaged salads, microscopic examination detected *T. gondii*‐like oocysts in 0.8% of samples, with numbers of oocysts ranging from 62 to 554/g of vegetables.^20^

We aimed to estimate the prevalence of *T. gondii*‐like oocyst shedding in the feral and owned cat population in Damascus, Syria.

## 2 MATERIALS AND METHODS

Fresh fecal samples were collected between October and December 2017 in Damascus and the surrounding countryside within 15 km of the city from 100 cats, consisting of 68 feral cats and 32 privately owned cats kept in houses. Fecal samples were stored in 10% formalin until the time of analysis, and then all were examined for *T. gondii*‐like oocysts by direct microscopic examination with and without sugar flotation (Sheather's flotation).^9^

For this concentration method, fecal samples were washed repeatedly using double‐distilled water. Oocyst flotation was carried out according to a previously described procedure.^21^ Sheather's solution, which is used for oocyst flotation, is composed of 106 g of glucose, 100 mL of double‐distilled water and 0.8 mL of liquid phenol, resulting in a specific gravity of 1.27. One volume of Sheather's solution was added to each fecal sample, vortexed briefly, and centrifuged for 15 min at 1000 × *g*. A wire loop (diameter = 6 mm) was used to touch the floating surface of the sample, and the sample was examined by microscopy at 400× magnification for sporulated (oocyst with 2 sporocysts containing 4 sporozoites each) or unsporulated (with sporont occupying most of the oocysts) *T. gondii*‐like oocysts.

### 2.1 Statistical analysis

Statistical analysis was performed using the SPSS 24 program, and the Chi‐squared test was used to assess differences in the prevalence of *T. gondii*‐like oocyst shedding in feral and owned cats. *P* < .05 was considered statistically significant.

## 3 RESULTS

Feces of 100 cats were examined by microscopy for detection of *T. gondii*‐like oocysts to assess oocyst shedding. Thirty‐six percent (36/100) of the samples contained *T. gondii*‐like oocysts, with feral cats showing higher levels of shedding than owned cats as shown in Table 1. The difference in shedding prevalence between feral cats (38.25%; 26/68) and owned domestic cats (31.2%; 10/32) was not significant (*P* = .66).

**TABLE 1:** Prevalence of *Toxoplasma gondii*‐like oocysts in feral and household cats.

	No.	Positive oocysts	%	*P*‐value
Feral cats	68	26	38.2	.66
Household cats	32	10	31.3	
Total	100	36	36	

## 4 DISCUSSION

Toxoplasmosis can be transmitted to humans by 3 principal routes. First, humans can eat raw or inadequately cooked meat. Second, humans can inadvertently ingest oocysts that cats have passed in their feces, either in a cat litter box or outdoors (eg, contaminated water, soil from gardening, unwashed fruits or vegetables). Third, a woman can transmit the infection to her unborn fetus.^22^

The ingestion of unfiltered water contaminated with *T. gondii* oocysts has been linked to toxoplasmosis.^23^ Thus, drinking water is increasingly being investigated as a risk factor, and has been found to be an important source of infection in tropical and subtropical countries, where surface water may be used for human consumption without any purification.^24^ Unwashed vegetables and fruits, or washing with contaminated water, also could increase risk of *T. gondii* infection. In addition, the role of water that may contain infective Toxoplasma oocysts and thereby contaminate fruit or vegetables during growth is important, as previously shown.^25^

Our aim was to determine the prevalence of oocyst shedding in cats in Damascus, Syria, where no oocyst shedding data have been reported previously. Examination of the collected samples showed that 36% (36/100) of the cats were shedding *T. gondii*‐like oocysts. In the coccidian group, there are 3 genera, including *Toxoplasma*, *Hammondia*, and *Neospora*, which are similar in shape and size, and difficult to differentiate without PCR.^26^ These genera were not differentiated in our study, which may explain the high prevalence found in our study. However, our results suggest that cats play an important role in the transmission of zoonotic diseases in Damascus, Syria.

Regarding Syria's neighboring countries, a single study from Beirut, Lebanon was found, and showed that 9.9% of cats in Beirut shed fecal *T. gondii*‐like oocysts.^27^ The composition of the diet of different groups of cats in different regions likely contributes to the differences in oocyst shedding reported. Studies from different countries on the prevalence of *T. gondii*‐like oocysts performed by examining fecal samples from cats showed the prevalence of infection in cats to be 2.1% of 240 in Kuwait,^28^ 9.1% of 4652 in Doha city,^29^ 23% of 100 in Nouakchott using the flotation method,^30^ 35.6% of 215 domestic cats in central and south Portugal,^31^ and 2.78% of 360 fecal samples in Henan and Beijing in central China examined using the same method as used in our study.^32^ Studies from 9 of Korea's largest cities and all regions of Sweden indicate that there is minimal risk of *T. gondii*‐like transmission from owned cats to humans.^33^, ^34^ A single study in southern Thailand using PCR showed that the prevalence of *T. gondii* oocysts was 4.7% of 205 fecal samples because PCR can differentiate genera and species of coccidia.^35^

Data on the frequency, severity, and duration of clinical signs of toxoplasmosis in humans and animals are crucial to understand the burden of the disease, which could lead to more adequate prevention strategies. To prevent food‐borne *T. gondii* infections, several precautions have been suggested.^22^, ^36^ Good hygiene practices are important, especially for pregnant women (e.g., frequent hand washing, wearing gloves when in contact with soil or feces from cats and other animals, boiling surface water before drinking to kill oocysts, cooking meat to safe temperatures, thoroughly washing or peeling fruits and vegetables before eating). Measures to prevent infection of food animals include keeping the animals indoors; keeping cats away from farms, feed, and bedding; providing clean drinking water and blocking access to surface water; and, implementing strict rodent control.^37^

Depending on environmental conditions (e.g., temperature, humidity), oocysts can survive for many years.^10^ Field studies are critical to evaluate the relative contributions of domestic and wild felids to environmental *T. gondii* oocyst burden on local, regional, and global scales.^38^, ^39^, ^40^

## 5 LIMITATIONS

Our study had some limitations. The number of samples was limited because of difficulties in sample collection because cats bury their feces. Other cities in Syria were not reachable because of the war. Therefore, future studies must include more samples from other cities. *Toxoplasma gondii* oocyst genera could not be differentiated in our study because PCR was not available at the microbiology laboratory in which the experiments were conducted.

Hence, future research on the seroprevalence of toxoplasmosis in cats living in Damascus are recommended to understand infection with *T. gondii* in feral and domestic cats, and assess its correlation with seroprevalence in humans.

## CONFLICT OF INTEREST DECLARATION

Authors declare no conflict of interest.

## OFF‐LABEL ANTIMICROBIAL DECLARATION

Authors declare no off‐label use of antimicrobials.

## INSTITUTIONAL ANIMAL CARE AND USE COMMITTEE (IACUC) OR OTHER APPROVAL DECLARATION

Authors declare no IACUC or other approval was needed.

## HUMAN ETHICS APPROVAL DECLARATION

Authors declare human ethics approval was not needed for this study.
